# Iodine Deficiency in Northern Paris Area: Impact on Fetal Thyroid Mensuration

**DOI:** 10.1371/journal.pone.0014707

**Published:** 2011-02-16

**Authors:** Dominique Luton, Corinne Alberti, Edith Vuillard, Guillaume Ducarme, Jean François Oury, Jean Guibourdenche

**Affiliations:** 1 Université Paris VII; AP-HP, GHU nord, Hôpital Beaujon, Service de Gynécologie Obstétrique, Clichy, France; 2 Université Paris VII; AP-HP, GHU nord, Hôpital Robert Debré, Service de Gynécologie Obstétrique, Paris, France; 3 Université Paris V; AP-HP, GHU ouest, Hôpital Cochin Port Royal, Service de Biochimie Hormonologie, Paris, France; 4 Université Paris VII; AP-HP, Hôpital Robert Debré, Unité d'Epidemiologie Clinique; Inserm, CIE 5, Paris, France; Ecole Normale Supérieure de Lyon, France

## Abstract

**Introduction:**

Iodine is essential for normal fetal and neonatal development. We studied the prevalence and impact on fetal thyroid development of iodine deficiency in pregnant women in the northern part of the Paris conurbation.

**Materials and Methods:**

110 patients underwent several determinations of urinary iodine excretion (UIE) and of serum FT4, FT3, and TSH. Fetal thyroid gland size was assessed using ultrasonography.

**Results:**

We found evidence of widespread iodine deficiency (mean UIE, 49.8 µg/L [standard deviation, 2.11]). Iodine deficiency did not correlate significantly with maternal thyroid parameters but showed a significant negative correlation with fetal thyroid gland size (rho = 0.25, *P* = 0.02).

**Conclusion:**

Iodine deficiency during pregnancy is still a problem in our geographical area and affects the fetal thyroid gland.

Clinical Trials.gov NCT00162539

## Introduction

During pregnancy, an appropriate supply of iodine is essential to maintain homeostasis in both the mother and the fetus [1]. Compared with maternal hypothyroidism, iodine deficiency may have an even greater impact on fetal neurocognitive development [2], [3], [4], probably because the area under the curve of fetal blood thyroxine concentrations is lower. The prevention, early detection, and immediate correction of iodine or thyroxine deficiency in pregnant women are high priorities.

Increased size of the neonatal thyroid gland measured using ultrasonography just after delivery has been reported in infants born to mothers with iodine deficiency [5]. We have many years of experience with ultrasonographic fetal thyroid gland measurements. We use both our own unpublished reference curves and the curves established by Ranzini et al. [6].

France is considered an area of moderate iodine deficiency [7], [8], [9]. To date, no consensus has been developed regarding the appropriateness of iodine supplementation before and during pregnancy.

The objective of the prospective observational study reported here was to assess the impact of maternal iodine status on fetal and neonatal thyroid gland size. We studied pregnant women living in the northern part of the Paris conurbation and their fetuses and neonates.

## Materials and Methods

### Subjects

We planned to recruit 110 pregnant patients receiving routine prenatal care at a single tertiary-level teaching hospital in northern Paris, France. Inclusion criteria were normal pregnancy, having signed the study informed consent document, and being covered by the French public healthcare insurance system. Exclusion criteria were the presence of chronic disease; iodine supplementation; current or past thyroid disease; fetal abnormalities; multiple pregnancy; pregnancy induced using assisted reproductive technology; and abnormal thyroid hormone concentrations at baseline.

**Figure 1:**
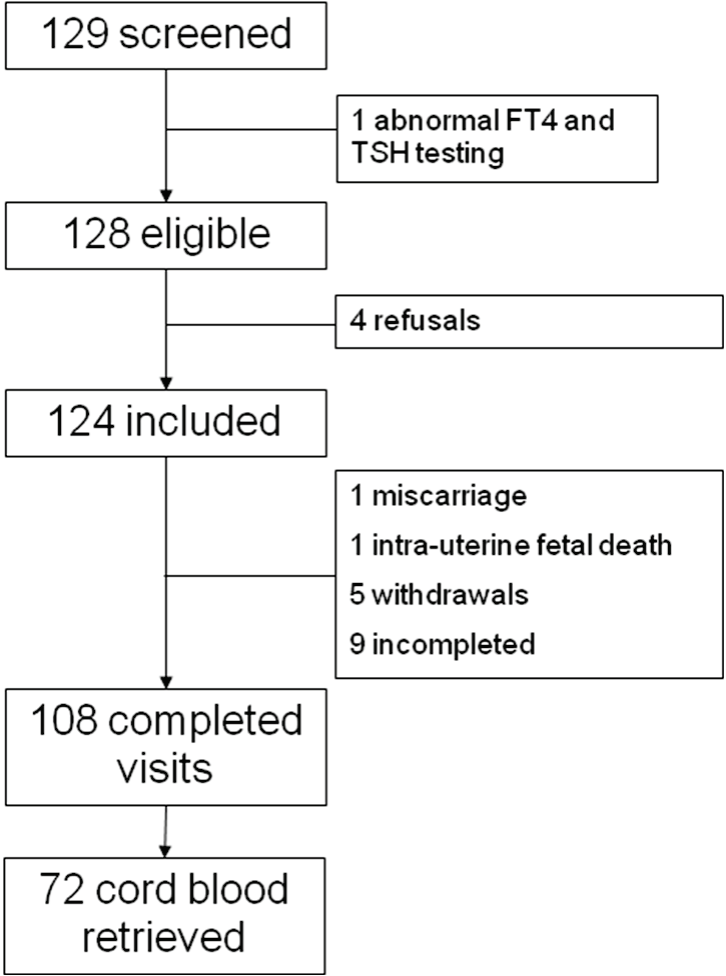
Flow-chart.

Of 129 patients who were invited to participate in the study 4 refused participation and 1 had abnormal serum thyroid hormone concentrations. Of the remaining 124 patients, 108 (87%) attended all the study visits. One patient had a miscarriage and another fetus died in utero at 22 weeks gestational age (WGA). Five additional patients asked to leave the study later during the pregnancy, usually at the request of the husband (Figure 1). None of the study patients delivered prematurely. Cord blood samples were obtained at delivery from 72 fetuses.

### Methods

Study data were collected at four time points: 12 WGA, 22 WGA, 32 WGA, and birth. At the inclusion visit at 12 WGA, a maternal blood sample was obtained for assays of free triiodothyronine (FT3), free thyroxine (F4), and TSH; as well as a urine sample or a 24 hours collection for determination of urinary iodine excretion (UIE). At both 22 and 32 WGA, ultrasonography of the fetal thyroid gland was performed for measurement of thyroid diameter and circumference. In addition, at 32 WGA a maternal blood sample was collected for assays of serum FT3, FT4, TSH, and iodine; as well as a urinary sample or a 24 hours collection for UIE measurement. Finally, at delivery maternal blood and cord blood samples were used for assays of FT3, FT4, and TSH. Ultrasonography of the thyroid gland was performed, and the results of the neonatal screening test for hypothyroidism were collected.

Ultrasonography was used to measure the diameter and circumference of the fetal or neonatal thyroid gland, as previously described [10], [11], [12], using an EVB 525 variable-focus ultrasound machine (Hitachi, Hialeah, FL, USA) with a 3.5-MHz sector transducer. To minimize variability in fetal thyroid gland diameter measurements due to differences in fetal size, we normalized fetal thyroid gland diameter for fetal head circumference.

All blood samples were tested after collection of all study data was complete. All sera were frozen at −80°C until use. UIE and serum iodine were assayed in Cerba laboratories using inductively coupled plasma-mass spectroscopy (Agilent 7500ce, Santa Clara, CA, USA). The limits of detection were 5 µg/L and 15 µg/L for serum and urine, respectively. Interassay variability was 7.8% in serum and 3.5% in urine; intraassay variability was always lower than interassay variability. UIE was expressed in µg/24 h when a 24-hour urine collection was obtained and in µg/L when a single urine sample was used. Single samples were always collected in the morning. Serum iodine was expressed in nanomole per liter.

Serum FT3, FT4, and TSH were assayed in our hospital laboratory on an ACS-180SE automate, using a chemiluminescent immunoassay. Variability was 3.3%, for FT3, 6.6%, for FT4, and 8.4% for TSH. The limits of detection were 0.3 pm/L, 1.3 pm/L, and 0.02 IU/L, respectively.

### Statistics

To detect a correlation coefficient of 0.3 between UIE and fetal thyroid size, with alpha set at 2.5% and beta at 10%, 110 patients were needed.

We previously established reference curves for fetal thyroid diameter and circumference in our geographic area (unpublished data). The values were similar to those reported by Ranzini et al [6]. Previous studies showed endemic iodine deficiency in France and established mean and median UIE values.

Quantitative variables were described as mean (SD) and qualitative variables as frequency (percentages). We computed Spearman's correlation coefficient to assess correlations linking maternal thyroid parameters (FT3, FT4, TSH), maternal iodine status (UIE and serum iodine), and fetal/neonatal thyroid size. All tests were two-tailed. Statistical analyses were performed using Statview Software V5 (SAS Institute Inc, Chicago, IL, USA; Copyright 1992–1998 Version 5.0.) *P* values less than 0.05 were considered significant.

The appropriate ethics committee (Comité de Protection des Personnes d'Ile de France) approved the study protocol on May 12, 2005, under the number 0511132.

## Results

**Figure 2:**
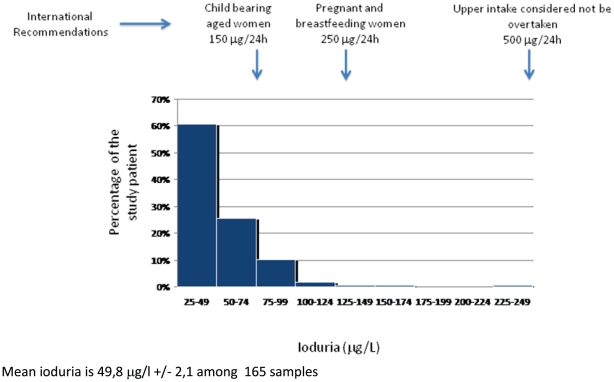
Urinary iodine excretion in pregnant women at 12 GA in the northern part of the Paris conurbation in 2006-2007 (pooled data). Mean ioduria is 49,8 µg/l +/− 2,1 among 165 samples.

Our UIE data obtained at 12 WGA, as well as the pooled UIE results, indicated iodine deficiency (Figure 2). Thus, the mean pooled value was 49.8 µg/L (SD, 2.11). UIE values were not significantly different between 12 WGA and 32 WGA (48.0 [SD, 2 µg/L] and 52.4 [SD 4 µg/L], respectively). UIE was determined on 24-hour urine collections in one-third of patients and single urinary samples in the remaining two-thirds. When we compared UIE values obtained using these two types of urine specimens, we found slightly higher values in the 24-hour collections (56.4 [SD, 2.8] µg/L) than in the single samples (44.2 [SD 2.5] µg/L); the difference was statistically significant (*P*<0.05) but was not clinically relevant.

Maternal thyroid hormone values at 12 GA were within the normal ranges for our laboratory (Table 1). As expected, FT3 and FT4 decreased from 12 to 32 WGA, whereas TSH increased (Table 1). These variations were not influenced by the UIE nor by the serum iodine.

**Table 1:** Changes in maternal serum levels of free triiodothyronine, free thyroxine, and TSH from 12 weeks' gestational age to delivery; cord blood values of the same parameters; and maternal serum iodine and urinary iodine excretion.

Maternal blood	Statistics	FT3 pmole/L	FT4 pmole/L	TSH IU/L	UIE µg/L	Serum iodine nmole/L
12 WGA	Mean (SD)	4.7 (0.4)	14.1 (1.49)	1.29 (0.63)	48.02 (2)	NA
	Min-MaxN	3.8–5.7116	10.4–18.2116	0.08–3.17116	30–117102	
32 WGA	Mean (SD)Min-MaxN	4.2 (0.4)3.3–5.396	12.1 (1.5)8.8–15.596	1.62 (0.73)0.46–4.596	52.4 (4)30–23363	695 (41)271–190076
Delivery	Mean (SD)	4.1 (0.6)	12.2 (1.7)	2.87 (1.5)	NA	NA
	Min-MaxN	1.5–5.285	8.7–16.785	0.22–9.885		
**Cord Blood**	Mean (SD)	2.53 (0.08)	14.1(0.2)	7.7 (0.8)	NA	NA
	Min-MaxN	1.5–4.772	9.5–19.272	0.5–2772		

FT3, free triiodothyronine; FT4, free thyroxine; TSH, thyroid-stimulating hormone; UIE, urinary iodine excretion; WGA, weeks' gestational age; NA, not available.

At 12 WGA, we found no significant correlations linking FT3, FT4, TSH, and UIE. A trend was found for high TSH, low T4, and low UIE. None of the FT3, FT4, or TSH values (12 WGA, 32 WGA, and delivery) correlated significantly with UIE, although a trend was noted (Table 2).

**Table 2:** Correlations between urinary iodine excretion at 12 weeks' gestational age (WGA) and serum thyroid hormone levels at 12 and 32 WGA.

		12 WGA			32 WGA		
		FT3	FT4	TSH	FT3	FT4	TSH
**Thyroid hormones,** **12 WGA**	FT3	1					
	FT4	0.17	1				
	TSH	−0.04	−0.19£	1			
**Thyroid hormones,** **32 WGA**	FT3	0.41*	−0.11	−0.24&	1		
	FT4	−0.15	0.30$	−0.24&	0.09	1	
	TSH	−0.13	−0.17	0.56*	−0.07	−0.25&	1
**UIE, µg/L, 12 WGA**		0.19£	0.09	−0.02	0.003	0.08	0.01

*p<0.001

$ *p* = 0.004

& *p* = 0.02

£ 0.05<*p*<0.10

All other *p* values were above 0.10.

FT3, free triiodothyronine; FT4, free thyroxine; TSH, thyroid-stimulating hormone; UIE, urinary iodine excretion; WGA, weeks' gestational age.

**Figure 3:**
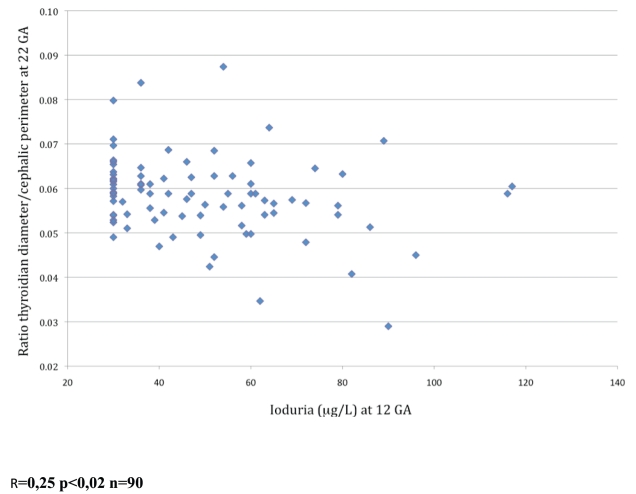
Correlation between maternal urinary iodine excretion at 12 weeks gestational age (WGA) and the ratio of fetal thyroid gland diameter over fetal head circumference at 22 weeks gestational age (n = 90). R = 0,25 p<0,02 n = 90.

Fetal thyroid gland size correlated significantly with UIE considered as a continuous variable or as a categorical variable (mild vs. severe iodine deficiency). Both the ratio of fetal thyroid gland diameter over fetal head circumference and UIE were available for 90 women. In these patients, the fetal thyroid gland diameter/head circumference ratio showed a significant negative correlation with UIE (rho =  −0.25; *P* = 0.02, Figure 3).

Mean maternal serum iodine at 32 WGA was 695 (SD, 41) nmole/L, which was normal according to international normative data [13]. Mean maternal serum iodine at 32 WGA did not correlate with mean maternal UIE at the same time point but correlated significantly with the fetal thyroid gland diameter/head circumference ratio (rho =  −0.28; *P* = 0.02) at 32 WGA, with maternal serum FT4 at 12 WGA (rho = 0.37; *P* = 0.001), and with maternal serum FT4 at 32 WGA (rho = 0.36; *P* = 0.02).

The neonatal screening test for hypothyroidism consisted in a TSH assay on capillary blood on postnatal day 3. The TSH concentration was below the limit of detection [15 µIU/ml) in all neonates. TSH, FT4, and FT3 concentrations in cord blood at delivery are reported in Table 1. Cord blood TSH correlated significantly with cord blood FT3 (rho =  −0.34; *P*<0.01) and maternal serum FT4 at 32 WGA (rho =  −0.26; *P*<0.05).

Neonatal thyroid gland measurements were obtained in only too few babies, which was not sufficient for a valid analysis.

## Discussion

The main findings from our prospective observational study in unselected pregnant women and their fetuses are that iodine deficiency is widespread in the geographic area served by our hospital and that lower maternal UIE values are associated with larger fetal thyroid gland size.

Iodine deficiency is a well-known public health problem in many parts of the world [14]. Moderate iodine deficiency is endemic in France, where few data are available regarding the impact of iodine deficiency on fetal development. Recent international guidelines indicate that the iodine needs during pregnancy are higher than previously thought. Although UIE data indicate iodine deficiency in our study population, only 1 of the 129 women screened for the study had abnormal serum thyroid hormone levels at 12 WGA. Thus, serum thyroid hormone assays do not seem useful for detecting iodine deficiency in pregnant women. Our hospital serves a low-income area where inadequate nutrition is common. Figure 2 indicates that 100 to 150 µg of supplemental iodine per day would not result in UIE values above the upper limit of the recommended range (500 µg/24 h) in most women.

We found no significant correlation between maternal serum thyroid hormone levels and UIE, in contrast to an earlier study [7]. One possibility is that our population was too homogeneous regarding the presence and severity of iodine deficiency to show such a correlation. As expected, serum FT3 and FT4 decreased and TSH increased over time; however, these three parameters were not significantly influenced by UIE. In a recent study conducted in Slovenia, in a place where the iodine supply is adequate, UIE showed no correlations with maternal serum TSH, FT4, or FT3 during pregnancy or after delivery [18]. However, in our study, maternal FT4 at 32 WGA showed a significant negative correlation with neonatal cord blood TSH, underlining the importance of maternal FT4 levels for the fetus.

Serum iodine is not considered a valid marker for iodine deficiency. In our study population, serum iodine levels were within the normal range, although UIE values indicated iodine deficiency. In addition, no significant correlation was found between serum iodine and UIE. Nevertheless, maternal serum iodine levels showed some correlations with fetal thyroid gland size and with maternal serum FT4, in keeping with earlier data.

We found a small difference between UIE values in single samples and 24-hour collections. We have no satisfactory explanation for this finding. However, the difference is too small to be clinically relevant and does not detract from our results.

A major finding from our study is that lower maternal UIE values were associated with a larger size of the fetal thyroid gland. Thyroid gland hypertrophy in response to iodine deficiency is well known to occur in adults and in pediatric patients, including neonates [5]. Our data indicate that the deleterious effect of iodine deficiency is already detectable during fetal life at least on the size of the thyroid gland. This is in accordance with what we expect to be a continuum of deleterious effect of iodine deficiency all life long including fetal period. Although the women in our study had iodine deficiency, the cord blood levels of FT3, FT4, and TSH were within the normal ranges. An earlier study found that maternal iodine deficiency was associated with increased neonatal TSH levels between postnatal days 3 and 4 [15]. The number of neonates who underwent ultrasonography of the thyroid gland was too small for an evaluation of the potential impact of maternal iodine deficiency on the neonatal thyroid gland.

Our findings indicate that iodine supplementation should be offered to all women of childbearing potential and to all pregnant women in our geographic area. Although iodine supplementation has been found beneficial even when started only in the second trimester of pregnancy [16], the benefits are greatest when it is begun before conception or very early during the pregnancy [17]. Consequently, ultrasonography of the fetal gland is probably not useful as a screening test for maternal iodine deficiency. Given the state of our knowledge, a randomized or comparative study of iodine supplementation would be unethical.
